# Inflammatory biomarker candidates in dementia with Lewy bodies: diagnostic classification, clinical associations, and survival

**DOI:** 10.21203/rs.3.rs-11368506/v1

**Published:** 2026-11-01

**Authors:** Ariana Pritha, Peter Swann, Maura Malpetti, Anna McKeever, Elijah Mak, George Savulich, Ajenthan Surendranathan, Owen Swann, Amanda Heslegrave, Henrik Zetterberg, James B. Rowe, Leonidas Chouliaras, John T. O’Brien

**Affiliations:** University of Cambridge School of Clinical Medicine; University of Cambridge School of Clinical Medicine; UK Dementia Research Institute; University of Cambridge School of Clinical Medicine; University of Cambridge School of Clinical Medicine; University of Cambridge School of Clinical Medicine; University of Cambridge School of Clinical Medicine; UK Dementia Research Institute; UK Dementia Research Institute; Sahlgrenska University Hospital; UK Dementia Research Institute; University of Cambridge School of Clinical Medicine; University of Cambridge School of Clinical Medicine

**Keywords:** Dementia with Lewy bodies, Blood biomarkers, Inflammatory panel

## Abstract

Dementia with Lewy bodies (DLB) is a common neurodegenerative dementia that remains difficult to distinguish clinically from Alzheimer’s disease (AD) and related disorders because of overlapping clinical features and frequent mixed pathology. Because alpha-synuclein pathology is associated with central and peripheral immune alterations, targeted measurement of inflammation-related biomarker candidates may provide a minimally invasive means of characterizing disease biology and supporting differential diagnosis. We examined whether plasma concentrations of proteins on the Olink Target 96 Inflammation panel differed between participants with DLB (n = 20), mild cognitive impairment and/or AD (MCI+/AD; n = 15), and cognitively normal controls (n = 15), and assessed their associations with clinical measures and survival. Differential-expression analyses, covariate-adjusted logistic regression with receiver operating characteristic (ROC) analysis, protein-clinical correlations, survival analyses, and pathway enrichment analyses were performed. No protein survived false-discovery-rate correction; nominal associations therefore describe exploratory signals. Relative to controls, DLB showed nominally higher CCL20 and CXCL11. Compared with MCI+/AD, DLB showed nominally lower MCP-2 and IL-2 and higher IL-18R1, TRANCE, GDNF, and IL-12B. AUCs derived from multivariable logistic models containing each protein and prespecified covariates ranged from 0.853 to 0.890 for the DLB versus MCI+/AD comparison. Higher TRANCE was nominally associated with greater motor severity (UPDRS: rho = 0.41, p = 0.0176), whereas its associations with ACE-R and MMSE did not reach nominal significance. Survival analyses identified limited and imprecise associations over a median follow-up of 6.5 years (range 0.07–9.80; IQR 3.49–7.93 years). Pathway analyses highlighted immune and intracellular signaling networks, including NF-kB and MAPK signaling. These exploratory findings support a targeted peripheral inflammatory signature in DLB that differs from normal ageing and MCI+/AD but require validation in larger independent cohorts.

## Introduction / Background

Dementia with Lewy Bodies (DLB) causes an uncomfortable lived experience for the patient and their family, made worse by the common delay or failure in diagnosis, especially in geriatric populations (Hogan et al., 2016). Defined by the accumulation of misfolded alpha-synuclein aggregates, DLB disrupts neuronal networks through mechanisms that extend beyond those of other neurodegenerative diseases. Clinically, it presents with fluctuating cognition, visual hallucinations, REM sleep behavior disorder, and Parkinsonism, often preceding or overshadowing memory decline (Pitton Rissardo et al., 2025). Yet in practice, these features rarely align cleanly and patients move through the healthcare system with variable and changing labels: Alzheimer’s disease, psychiatric illness, or Parkinson’s disease, before DLB is considered (Ghaly & Markopoulou, 2025). This diagnostic instability is not incidental and reflects a deeper biological overlap, where more than half of DLB cases harbor concurrent Alzheimer’s pathology, blurring both clinical and molecular boundaries (Ting et al., 2025; Schumacher et al., 2021). What remains missing is a signal that cuts across this noise early enough to matter. Neuroinflammation offers one such signal. The inflammatory landscape in DLB is not simply weaker or stronger than in Alzheimer’s disease, but rather, it is qualitatively different (Amin, Holmes, et al., 2020). Alpha-synuclein aggregation activates microglia, but this activation appears restrained, even adaptive (Chung et al., 2022; Deyell et al., 2023). Post-mortem analyses consistently show elevated markers of phagocytic microglia, including HLA-DR and CD68, alongside increased CD16 expression. At the same time, reduced CD32a signaling suggests a shift away from pro-inflammatory amplification toward regulated immune engagement (Amin, Holmes, et al., 2020).

This stands in contrast to Alzheimer’s disease, where amyloid-β and tau deposition drive a more sustained and damaging inflammatory response (Ismail et al., 2020). Microglial activation in AD correlates strongly with protein burden, contributing directly to synaptic loss and neuronal death. DLB, by comparison, seems to operate within a narrower inflammatory window as one that may initially support clearance mechanisms before eventually failing (Abdulkhaliq et al., 2026; Van Wetering et al., 2024).

It remains challenging to make this distinction from peripheral assays. Peripheral cytokines, including IL-6, IL-1β, and TNFα, are elevated early in DLB, particularly during prodromal stages such as MCI-LB. Interestingly, these levels tend to decline as the disease progresses, implying a transient inflammatory surge rather than a sustained response (Amin, Boche, et al., 2020; Surendranathan et al., 2018). This temporal pattern differs from Alzheimer’s disease, where inflammatory markers often remain persistently elevated in parallel with accumulating pathology. Glial-derived proteins such as GFAP and YKL-40 further refine this picture. While both conditions show elevations, their magnitude and trajectory appear to differ, especially in the presence or absence of amyloid co-pathology (Mavroudis et al., 2021; Wennström et al., 2015). In DLB, these markers may reflect astrocytic adaptation rather than overt neurotoxicity. Taken together, these circulating signals suggest that the peripheral immune system is not merely reactive.

The difficulty in distinguishing DLB from MCI+/AD is greater in early stages, where both conditions converge clinically (McKeith et al., 2020). Patients may present with attention deficits, executive dysfunction, or mild cognitive impairment without clear defining features. Memory, often the anchor of Alzheimer’s diagnosis, may remain relatively preserved in DLB, but this distinction is subtle and easily overlooked (Kang et al., 2023). Compounding this, symptom fluctuation, which is a hallmark of DLB, undermines snapshot-based clinical assessments. A patient may appear cognitively intact during one visit and profoundly impaired during another. Standard screening tools fail to capture this variability (Matar et al., 2020).

Biomarkers offer a potential way out of this problem. Current approaches, including amyloid and tau imaging, struggle in cases of mixed pathology (Amin, Holmes, et al., 2020; Vrillon et al., 2024). Our group and others previously demonstrated that “AD biomarkers” are often also elevated in DLB (Chouliaras et al., 2022), and do not offer clear differentiation between the conditions (Malpetti et al., 2025). A blood-based inflammatory profile, however, may retain discriminatory power precisely because it captures how the disease behaves, not just what proteins are present. The potential of inflammatory biomarkers lies in their accessibility. Blood-based assays are scalable, repeatable, and capable of capturing longitudinal change. This is particularly relevant for DLB, where early diagnosis could meaningfully alter management, avoiding harmful medications, optimizing symptomatic treatment, and enabling targeted clinical trial enrollment.

More importantly, these markers may allow us to identify DLB before it fully manifests. The presence of an early, transient inflammatory signal suggests a window where intervention could disrupt disease progression, particularly in individuals with REM sleep behavior disorder or subtle cognitive changes. The goal of this scientific work was to further assess whether we could meaningfully incorporate the blood biomarker analyses to identify, in an exploratory manner, which inflammatory markers may provide us with some sort of insight into differentiating DLB patients uniquely from other groups.

Our previous study of the same NIMROD cohort used the Olink Target 96 Metabolism panel and identified metabolism-related biomarker candidates that differed between DLB, controls, and MCI+/AD (Pritha et al., 2026). The present study addresses a distinct, prespecified biological question by focusing on inflammation-related proteins, motivated by evidence of central and peripheral immune alterations in DLB (Amin, Holmes, et al., 2020; Surendranathan et al., 2018). We hypothesized that DLB would show a targeted peripheral inflammatory biomarker profile distinct from both cognitively normal ageing and MCI+/AD. We further explored whether individual inflammation-related proteins were associated with cognition, motor severity, and survival; these analyses were considered exploratory rather than confirmatory.

## Methodology

The detailed protocols are adapted from a previous paper published by the group on the Olink 96 Target Metabolism panel assay with some variations (Pritha et al., 2026).

### Participants and clinical data

The NIMROD study cohort provided us with the participant data, outlined in previously published work (Bevan-Jones et al., 2017), and participants were stratified into mild cognitive impairment or Alzheimer’s disease (MCI+/AD) or dementia with Lewy bodies (DLB) groups based on current clinical diagnostic criteria with specifics outlined in National Institute on Aging-Alzheimer’s Association Criteria and 2005/2017 McKeith consensus criteria for DLB (McKeith et al., 2017). Individuals classified as amyloid-positive mild cognitive impairment (MCI) met clinical criteria for MCI characterized by subjective cognitive concerns corroborated by objective testing, with preserved independence in daily functioning. Amyloid burden was determined using Pittsburgh Compound B positron emission tomography (PiB-PET), and participants were considered positive if values exceeded a centiloid threshold of 19.

### Clinical assessments

All participants underwent an extensive baseline assessment encompassing neurological examination, cognitive testing, and neuropsychiatric evaluation in line with established study protocols. Participants were subsequently re-evaluated at yearly intervals for a maximum of three years within the NIMROD cohort (Bevan-Jones et al., 2017). Global cognitive status was quantified using the Addenbrooke’s Cognitive Examination-Revised (ACE-R) (Mioshi et al., 2006).

Mortality outcomes were ascertained through linkage with electronic healthcare records for individuals in the DLB and MCI+/AD groups. Across these cohorts, 22 deaths were recorded during the observation period. The demographics were previously published in the Olink Metabolism Panel paper by our group, and the same demographic was used for this Olink Inflammatory Panel (Table 1).

### Blood collection and storage

Approximately 80 mL of venous blood was obtained from each participant. Samples were processed by centrifugation to isolate plasma and serum fractions, aliquoted, and preserved at − 70°C until analysis.

### Proteomic profiling and biomarker quantification

Plasma samples from participants with DLB (n = 20), MCI+/AD (n = 15), and cognitively normal controls (n = 15) were analyzed using the Olink Target 96 Inflammation panel (Olink Proteomics, Uppsala, Sweden), which measures 92 protein targets (listed in Supplementary File 1 and on the Olink website). Assays were conducted at the UK Dementia Research Institute Biomarker Factory at University College London. Olink results were reported as normalized protein expression (NPX; log2 scale) and standardized within the full cohort for statistical modelling where specified.

Genotyping and construction of genetic risk scores

Genome-wide genotyping was performed using the Illumina OmniExpress-24 v1.3 array platform, with genotyping quality control conducted as described previously (McKeever et al., 2025). Lewy body dementia (LBD) genetic risk scores were constructed from five genome-wide significant variants reported in the LBD genome-wide association study by Chia et al. (2021).Each genetic risk score was calculated as the weighted sum of risk alleles, using the published effect estimates as weights. A second LBD genetic risk score excluded variants within the *APOE* region to represent non-*APOE* genetic susceptibility. *APOE*-associated risk was also represented separately as the count of *APOE* epsilon2 and epsilon4 alleles, weighted by their published effect estimates. All genetic risk variables were standardized within the study population before downstream analyses.

### Bioinformatics analyses

#### Pre-processing & proteomic profiling

Plasma inflammatory proteins were measured using the Olink proximity extension assay platform and reported as normalized protein expression (NPX; log_2_ scale). Clinical and demographic data, including diagnosis (Control, DLB, MCI+/AD), age, sex, levodopa dose, storage duration, and cognitive measures (MMSE, ACE-R, UPDRS), were merged by participant ID. Analyses were restricted to individuals with complete data for variables included in each model.

#### Data preprocessing

Proteins were retained if ≥ 50% of observations were non-missing and showed nonzero variance. Missing values were imputed using within-protein medians where required. For differential expression analyses, NPX values were quantile normalized and low-variance proteins (bottom 10%) were excluded. For correlation analyses, protein values were z-score scaled.

#### Differential expression analysis

Differential protein expression between diagnostic groups (DLB vs Control; DLB vs MCI+/AD) was assessed using linear models with empirical Bayes moderation (limma). Models included age, age^2^, sex, levodopa dose, and storage duration as covariates. Log_2_ fold changes and moderated P-values were computed, with false discovery rate (FDR) correction applied using the Benjamini–Hochberg method. Results were visualized using volcano plots.

#### Diagnostic classification (ROC analysis)

Biomarker performance was evaluated using covariate-adjusted logistic regression models of the form:

diagnosis~protein+age+sex+levodopadose+storageduration.

Predicted probabilities from these multivariable models were used to generate receiver operating characteristic (ROC) curves and calculate areas under the curve (AUCs). The reported AUCs therefore reflect each protein in combination with the prespecified covariates, rather than the protein alone.

#### Protein–clinical associations

Associations between protein levels and clinical measures (MMSE, ACE-R, UPDRS) were assessed using Spearman rank correlations. For adjusted analyses, linear regression models were fitted including age, sex, diagnosis, and storage duration as covariates. Multiple testing correction was applied within each outcome using the Benjamini–Hochberg method. Results were visualized using heatmaps and representative scatterplots.

#### Survival analysis

Time-to-event associations were evaluated using Cox proportional hazards models with protein levels as predictors and age, sex, diagnosis, and storage duration as covariates. Hazard ratios (HRs) with 95% confidence intervals were reported and visualized as forest plots.

#### Pathway enrichment analysis

Differentially expressed proteins were analyzed for pathway enrichment using over-representation analysis with FDR correction using the ShinyGo V 0.85 platform (Ge et al., 2020). Pathways were grouped into functional categories, and results were visualized using bubble plots, with point size representing overlap and color indicating − log_10_(FDR).

#### Statistical analysis

All tests were two-sided. Benjamini-Hochberg false-discovery-rate (FDR)-adjusted p < 0.05 defined statistical significance. Because no differential-expression or protein-clinical association survived FDR correction, unadjusted p < 0.05 findings are explicitly described as nominal and exploratory. Analyses were performed in R (v4.3) using limma, pROC, survival, ggplot2, and related packages. The prespecified analysis sequence evaluated DLB versus controls, DLB versus MCI+/AD, associations between inflammatory proteins and MMSE, ACE-R, and UPDRS, and Cox models incorporating clinical, genetic, and molecular variables.

## Results

### DLB vs. Control has modest differences in inflammatory protein levels

No protein survived FDR correction in the DLB versus control comparison. At the nominal threshold, CCL20 (log2FC = 0.71, p < 0.03) and CXCL11 (log2FC = 0.57, p < 0.03) were upregulated (Fig. 1A). Covariate-adjusted logistic models yielded AUCs of 0.923 for CCL20 and 0.920 for CXCL11 (Fig. 1B); these AUCs represent the protein-plus-covariate models rather than the proteins alone.

### DLB vs. MCI+/AD has key signature changes in inflammatory protein levels

No protein survived FDR correction in the DLB versus MCI+/AD comparison. At the nominal threshold, MCP-2 (log2FC = −0.72, p < 0.02) and IL-2 (log2FC = −0.30, p < 0.03) were lower in DLB, whereas IL-18R1 (log2FC = 0.45, p < 0.007), TRANCE (log2FC = 0.45, p < 0.03), GDNF (log2FC = 0.28, p < 0.04), and IL-12B (log2FC = 0.64, p < 0.03) were higher (Fig. 2A). Covariate-adjusted logistic models yielded AUCs of 0.890 for MCP-2 and IL-18R1, 0.873 for IL-2 and IL-12B, 0.863 for GDNF, and 0.853 for TRANCE (Fig. 2B). These AUCs reflect each protein together with age, sex, levodopa dose, and storage duration. Exploratory pathway analysis mapped these nominal protein signals to immune and signaling pathways including NF-kappaB, MAPK, extrinsic apoptosis, and PI3K-Akt (Fig. 2C). Results for MCI+/AD versus controls are provided in Supplementary File 1.

### Integrating protein expression levels with clinical scores

Across the full panel, most protein correlations with global cognitive scores (MMSE and ACE-R) were weak and did not reach nominal significance (Fig. 3A). TRANCE showed nominal inverse correlations with ACE-R (rho = −0.25, p = 0.0789) and MMSE (rho = −0.24, p = 0.088), neither of which met the nominal p < 0.05 threshold. Higher TRANCE was nominally associated with greater motor severity measured by UPDRS (rho = 0.41, p = 0.0176; Fig. 3B). Because UPDRS contributed to clinical group characterization and was concentrated in the DLB group, this association should not be interpreted as independent diagnostic evidence.

Associations of inflammatory proteins, cognition, and genetic risk with survival

Cox proportional-hazards models were used to estimate associations between protein expression and survival while including MMSE and the LBD genetic risk score excluding the APOE region as covariates (Fig. 4A–B). These variables were included for covariate adjustment; the analysis was not designed to test incremental discrimination or to establish that adding genetic risk improved survival prediction. APOE-specific and alternative genetic-risk specifications were not included in the primary model and should be reported separately if those sensitivity analyses are undertaken.

Most protein hazard-ratio estimates were close to the null and imprecise (Fig. 4A). In the DLB versus control model, TNFRSF9 had a markedly larger estimate than the other proteins, with a wide confidence interval.

When focusing on DLB vs MCI+/AD comparisons (Fig. 4B), a more consistent pattern emerged: CD40, SCF, CXCL11, IL7, and ADA showing moderate positive associations with risk. While these effects remained modest and confidence intervals were wide, the overall trend suggests a shift toward increased risk with higher levels of immune-related proteins. In contrast, IL-4 was the only marker showing a negative association, indicating a potential protective effect consistent with its anti-inflammatory role.

Overall, the adjusted survival associations were limited and less consistent than the cross-sectional classification signals. The models therefore provide exploratory estimates of association and should not be interpreted as validated prediction models.

## Discussion

We identified a narrow chemokine signal distinguishing DLB from controls at the nominal threshold: CCL20 (log2FC = 0.71, p < 0.03) and CXCL11 (log2FC = 0.57, p < 0.03) were higher in DLB (Fig. 1A). Neither association survived FDR correction. The direction and specificity of these exploratory signals are consistent with a targeted alteration in inflammatory signaling rather than a global shift across the assayed biomarker candidates.

Both CCL20 and CXCL11 are chemokines that sit upstream of immune cell recruitment, and their biology fits closely with what is known about early neuroinflammatory changes in DLB. CCL20, through CCR6 signaling, drives recruitment of Th17 cells and immature dendritic cells, which are not typically dominant in homeostatic CNS conditions (Koper et al., 2018; Ma et al., 2024). One interpretation could be that CCL20 reflects a compensatory or early-stage immune response, where immune trafficking across the blood–brain barrier is increased even in the absence of a classical cytokine storm.

CXCL11, on the other hand, reflects a slightly different axis of inflammation. As part of the CXCR3 ligand family, it is more closely tied to IFN-γ–mediated signaling and recruitment of activated T cells and NK cells (Ma et al., 2024; Nafeli Shahrestani et al., 2026). While this pathway is strongly amplified in conditions like AD or multiple sclerosis, its presence here, alongside CCL20, suggests that DLB may still engage Th1-type immune signaling, but in a more constrained or region-specific way. This aligns with the idea that DLB pathology involves disruption of white matter integrity and blood–brain barrier permeability, where even modest increases in CXCL11 could have downstream effects on immune cell infiltration and neuronal stress.

From a functional standpoint, both chemokines have been linked to processes that directly impact cognition. CCL20 has been associated with microglial activation and impaired neurogenesis, particularly in hippocampal regions, while CXCL11 contributes to a pro-inflammatory environment that can reduce synaptic density and worsen neuronal resilience over time (Nafeli Shahrestani et al., 2026; Wang et al., 2024). Though Fig. 1 focuses on case–control differences rather than cognition directly, the directionality of these markers is consistent with pathways known to contribute to cognitive decline across neurodegenerative diseases.

The ROC analysis provides an exploratory assessment of classification in this cohort. Multivariable models containing CCL20 or CXCL11 together with age, sex, levodopa dose, and storage duration yielded AUCs greater than 0.9 (Fig. 1B). These values should not be interpreted as the performance of either protein alone, and internal estimates from this small sample are likely optimistic without independent validation.However, CCL20 and CXCL11 are not disease-specific and are elevated across a range of inflammatory and vascular conditions. In DLB specifically, their elevation may reflect a unique immune imbalance rather than simply increased inflammation, which could explain why they separate DLB from controls despite overlapping with pathways seen in AD and other disorders.

In contrast to the relatively focused chemokine signal observed in the DLB versus control comparison, Fig. 2 reveals a more complex and bidirectional pattern when DLB is compared to MCI+/AD. What stands out is that the separation is not driven by a single pathway, but by a more complex shift involving both suppression and activation of distinct immune and neurotrophic processes. MCP-2 (log2FC=−0.72, p < 0.02) and IL-2 (log2FC=−0.30, p < 0.03) are reduced in DLB, and IL-18R1 (log2FC = 0.45, p < 0.007), TRANCE (log2FC = 0.45, p < 0.03), GDNF (log2FC = 0.28, p < 0.04), and IL-12B (log2FC = 0.64, p < 0.03) are elevated (Fig. 2A), suggesting that DLB occupies a different immunological state rather than simply representing a weaker version of AD-related inflammation.

The downregulation of MCP-2 and IL-2 is particularly interesting in this context (Fig. 2A). Both are involved in classical immune activation: MCP-2 in monocyte recruitment and IL-2 in T-cell proliferation which are processes that are typically upregulated in MCI and AD as part of a more overt inflammatory response (Iba et al., 2020; Liang et al., 2021). Their reduction in DLB suggests that this pathway is not just absent, but actively dampened. One interpretation is that DLB reflects a state of immune exhaustion or regulatory dominance, where adaptive immune signaling is suppressed, potentially limiting monocyte trafficking and altering how pathological proteins are cleared (Qiu et al., 2025). This fits with the broader idea that DLB pathology is less driven by classical cytokine amplification and more by dysregulated immune balance, which may allow α-synuclein pathology to propagate under conditions of insufficient immune surveillance (King et al., 2018).

At the same time, the upregulated markers point toward a compensatory or alternative inflammatory profile. IL-18R1 suggests increased sensitivity to IL-18 signaling, which can amplify IFN-γ–mediated responses even without large increases in ligand levels (Costantini et al., 2024; Grasso et al., 2026). IL-12B further supports a shift toward Th1/Th17 polarization, but in a way that appears more controlled than the widespread activation seen in AD (Oberstein et al., 2018). These markers suggest that DLB does not lack inflammation, but rather redistributes it toward pathways that favor immune priming over full activation (Yacoubian et al., 2025) (Fig. 2C).

TRANCE (RANKL) and GDNF introduce an additional layer that is not purely inflammatory (Fig. 2A, 3B). TRANCE is involved in immune cell differentiation and osteoimmune signaling (Wong et al., 1999), and its elevation may reflect broader systemic involvement, including comorbid processes that are more prominent in DLB. GDNF, on the other hand, is a neurotrophic factor, and its upregulation likely represents a compensatory response to ongoing neurodegeneration, particularly in dopaminergic systems (Chmielarz et al., 2020). This is consistent with the clinical phenotype of DLB, where motor and cognitive features overlap, and suggests that peripheral biomarkers may be capturing both injury and attempted repair.

From a diagnostic standpoint, this combination of decreased MCP-2/IL-2 and increased IL-18R1/TRANCE/GDNF/IL-12B provides a clearer separation between DLB and MCI+/AD than single markers alone. The ROC analysis supports this, with multiple proteins achieving high classification performance (Fig. 2B). Importantly, this pattern differs from what is typically observed in AD (Brosseron et al., 2014), where markers of generalized inflammation tend to dominate.

Important to note, the downregulation of IL-2 and MCP-2 may vary depending on whether patients are in early or advanced disease, and compensatory markers like GDNF are likely dynamic over time. The data suggests that DLB can be distinguished from MCI+/AD based on a distinct peripheral immune signature characterized by reduced classical immune activation and concurrent engagement of neurotrophic and Th1-associated pathways.

Among the inflammation-related proteins examined (Fig. 3A), TRANCE was highlighted because its direction of association was similar across cognitive and motor measures. Higher TRANCE was associated with lower ACE-R (rho = −0.25, p = 0.0789) and MMSE (rho = −0.24, p = 0.088), but neither cognitive association reached nominal significance. Its positive association with UPDRS met the nominal threshold (rho = 0.41, p = 0.0176; Fig. 3B), although no protein-clinical association survived FDR correction. TRANCE should therefore be viewed as an exploratory candidate for validation rather than an established clinical correlate.

In contrast to the classification analyses, the survival models showed limited and inconsistent associations across the inflammation-related proteins (Fig. 4). Most estimates were close to the null and confidence intervals were wide, indicating that this study does not establish individual inflammatory proteins as prognostic biomarkers.

TNFRSF9 was an outlying estimate in the DLB versus control survival model (Fig. 4A), with a log hazard ratio exceedingly approximately 4 and a wide confidence interval. Given the small cohort and limited number of events, the magnitude is more plausibly interpreted as model instability or sparse-data sensitivity than as a precise biological effect. Replication in a larger cohort is required before any prognostic interpretation.

In the DLB vs MCI+/AD comparisons, most markers show modest positive associations with outcome, with effect sizes clustering on the positive side of the distribution (Fig. 4B). This suggests that disease progression rather reflects a broader shift toward low-grade immune activation. Several proteins, including CD40, SCF, and CXCL11, show relatively larger effect sizes, although these are accompanied by wide confidence intervals, indicating substantial uncertainty. Despite this, the consistency in direction across multiple immune-related markers supports the idea that peripheral immune signaling contributes to progression in a distributed manner rather than through a single dominant mechanism. IL-4 stands out as the only marker with a negative association, suggesting a potential protective role. Given its established function as an anti-inflammatory cytokine, this finding aligns with the broader pattern of immune imbalance, where reduced anti-inflammatory signaling may contribute to worsening outcomes. Some of these markers, such as CXCL11, were also identified in earlier analyses as distinguishing between diagnostic groups, suggesting partial overlap between pathways involved in disease state and those influencing progression.

## Conclusion

In this cohort, DLB was associated with an exploratory profile of inflammation-related biomarker candidates that differed from both controls and MCI+/AD. Relative to controls, the nominal signal was limited to CCL20 and CXCL11; relative to MCI+/AD, nominal differences included lower MCP-2 and IL-2 and higher IL-18R1, TRANCE, GDNF, and IL-12B. None survived FDR correction, indicating that the pattern should be treated as hypothesis-generating rather than definitive. Survival analyses showed small and variable effects, and although TNFRSF9 appeared prominent in the combined model, this likely reflects instability related to the limited number of events rather than a reliable signal. More generally, proteins that performed well for distinguishing groups did not translate to strong predictors of survival. These findings should be interpreted with caution given the small sample size, which likely contributes to variability in effect estimates. Larger studies will be needed to confirm these patterns and determine whether these markers have practical value for disease stratification or prognosis.

## Supplementary Material

This is a list of supplementary files associated with this preprint. Click to download.

- SupplementaryFile1.xlsx

## Figures and Tables

**Figure 1:**
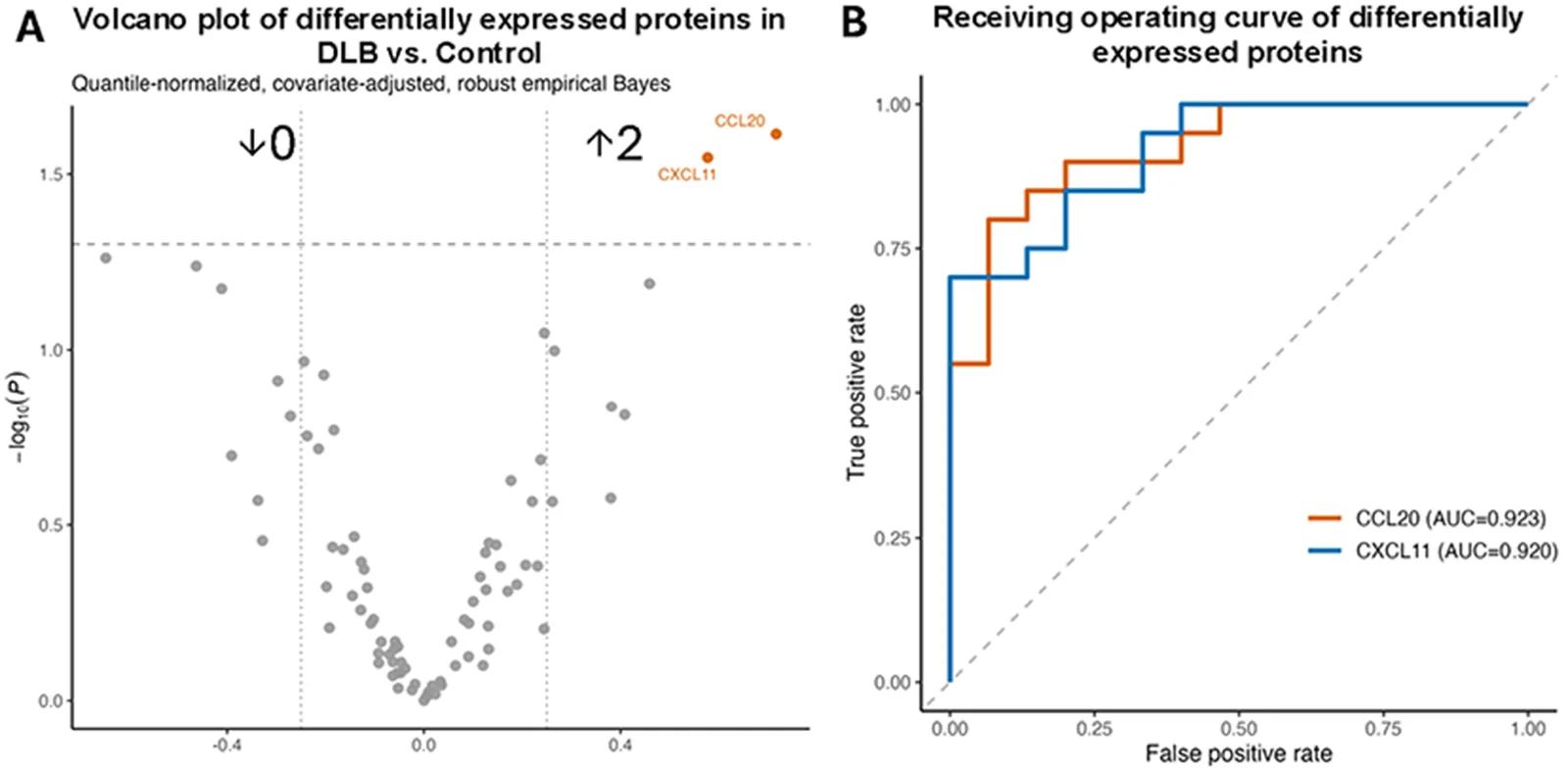
**(A)** The volcano plot contrasting DLB with control has thresholds of log2FC>|0.25| and p-value<0.05, demonstrating 0 downregulated proteins, and 2 upregulated proteins. Volcano plot of quantile-normalized, covariate-adjusted protein expression differences analyzed using a robust empirical Bayes framework. Each point represents an individual analyte, plotted by log_2_ fold change and statistical significance (-log_10_ P). Vertical dashed lines denote fold-change thresholds, and the horizontal dashed line indicates nominal significance. **(B)** Receiver operating characteristic (ROC) curves evaluating the discriminatory performance of CCL20 and CXCL11 following covariate adjustment. Both markers demonstrated excellent discriminatory performance, with CCL20 yielding an AUC of 0.923 (95% CI 0.838–1.000) and CXCL11 yielding an AUC of 0.920 (95% CI 0.836–1.000). The diagonal dashed line represents chance-level performance. For the DLB vs control comparison, the ROC shows the 2 upregulated proteins’ AUCs and ability to differentiate the condition.

**Figure 2:**
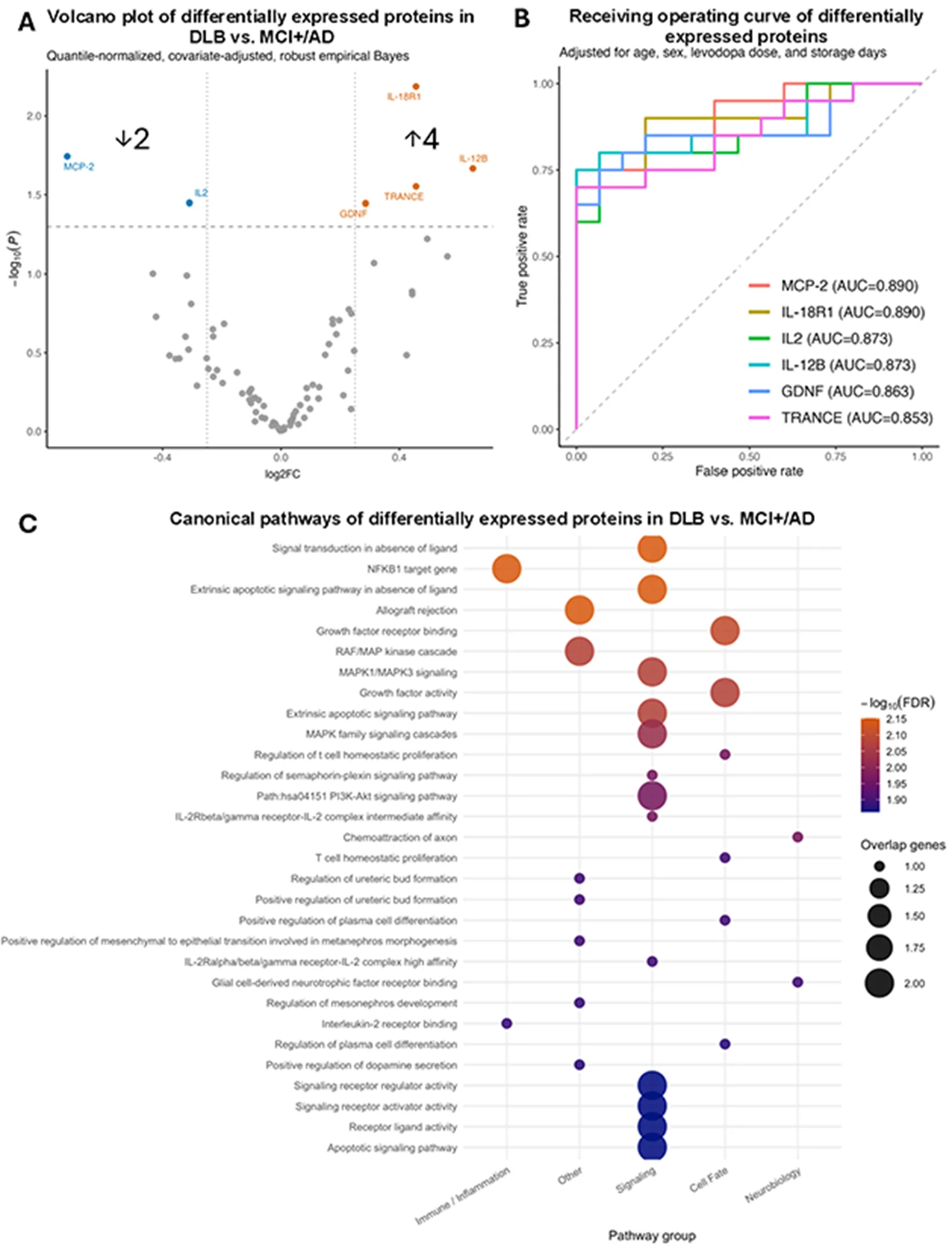
**(A)** The volcano plot contrasting DLB with MCI+/AD has thresholds of log2FC>|0.25| and p-value<0.05, demonstrates 2 downregulated proteins, and 4 upregulated proteins. **(B)** Receiver operating characteristic (ROC) curves for top-performing biomarkers following adjustment for age, sex, levodopa dose, and sample storage duration. ROC analyses demonstrated good discriminatory performance for all six proteins, with MCP-2 (AUC = 0.890, 95% CI 0.784–0.996) and IL-18R1 (AUC = 0.890, 95% CI 0.780–.000) achieving the highest classification accuracy, followed by IL-2 (AUC = 0.873, 95% CI 0.756–0.990), IL-12B (AUC = 0.873, 95% CI 0.750–0.997), GDNF (AUC = 0.863, 95% CI 0.735–0.992), and TRANCE (AUC = 0.853, 95% CI 0.727–0.979). A diagonal dashed line indicates chance performance. The six differentially expressed proteins from the previous analysis for DLB vs. MCI+/AD are represented with the ROC curves. **(C)** Canonical pathways from the 6 DE-proteins from DLB vs MCI+/AD comparison were used to identify pathways of potential interest to understand the underlying mechanistic changes in which the inflammatory proteins are involved. Pathway enrichment analysis of differentially expressed proteins, grouped by functional categories (immune/inflammation, signaling, cell fate, and neurobiology). Dot size reflects the number of overlapping genes per pathway, and color indicates statistical significance (-log_10_ FDR). Enriched pathways include NF-κB target gene signaling, MAPK cascade activity, extrinsic apoptotic signaling, and PI3K–Akt signaling, alongside immune-regulatory processes such as IL-2 receptor signaling and T cell homeostasis.

**Figure 3:**
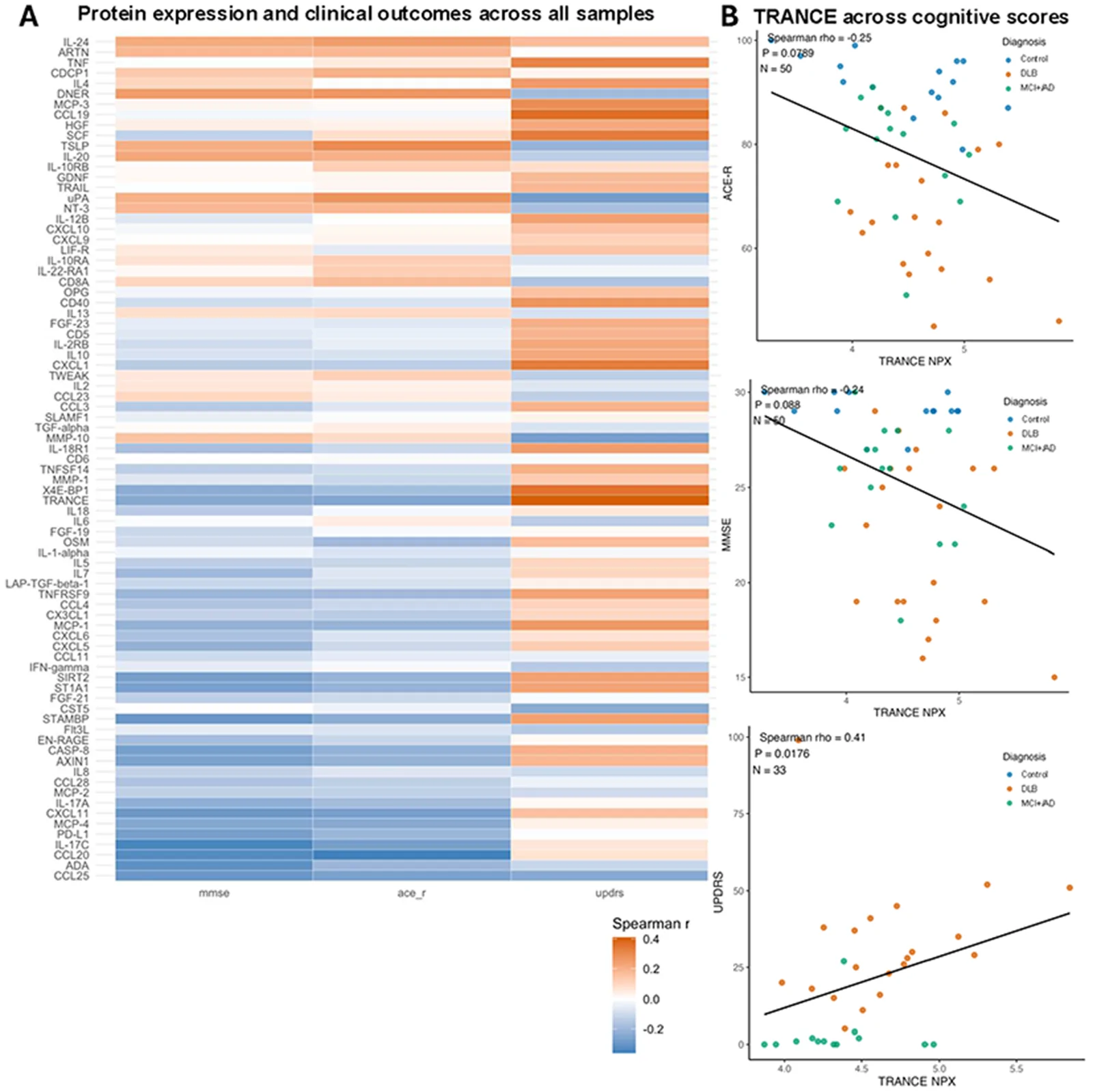
(A) Heatmap depicting Spearman correlation coefficients (rho) between plasma inflammatory proteins and clinical measures, including global cognition (MMSE and ACE-R) and motor severity (UPDRS). Rows represent individual analytes and columns represent clinical outcomes. The color scale indicates direction and magnitude of association (blue, negative; red, positive). (B) Scatterplots illustrating associations between TRANCE (TNFSF11) levels (NPX, log2 scale) and clinical outcomes. Each point represents one participant and is colored by diagnostic group. TRANCE was not nominally associated with ACE-R (rho = −0.25, p = 0.0789, n = 50) or MMSE (rho = −0.24, p = 0.088, n = 50), but was nominally positively associated with UPDRS (rho = 0.41, p = 0.0176, n = 33). No protein-clinical association survived FDR correction.

**Figure 4:**
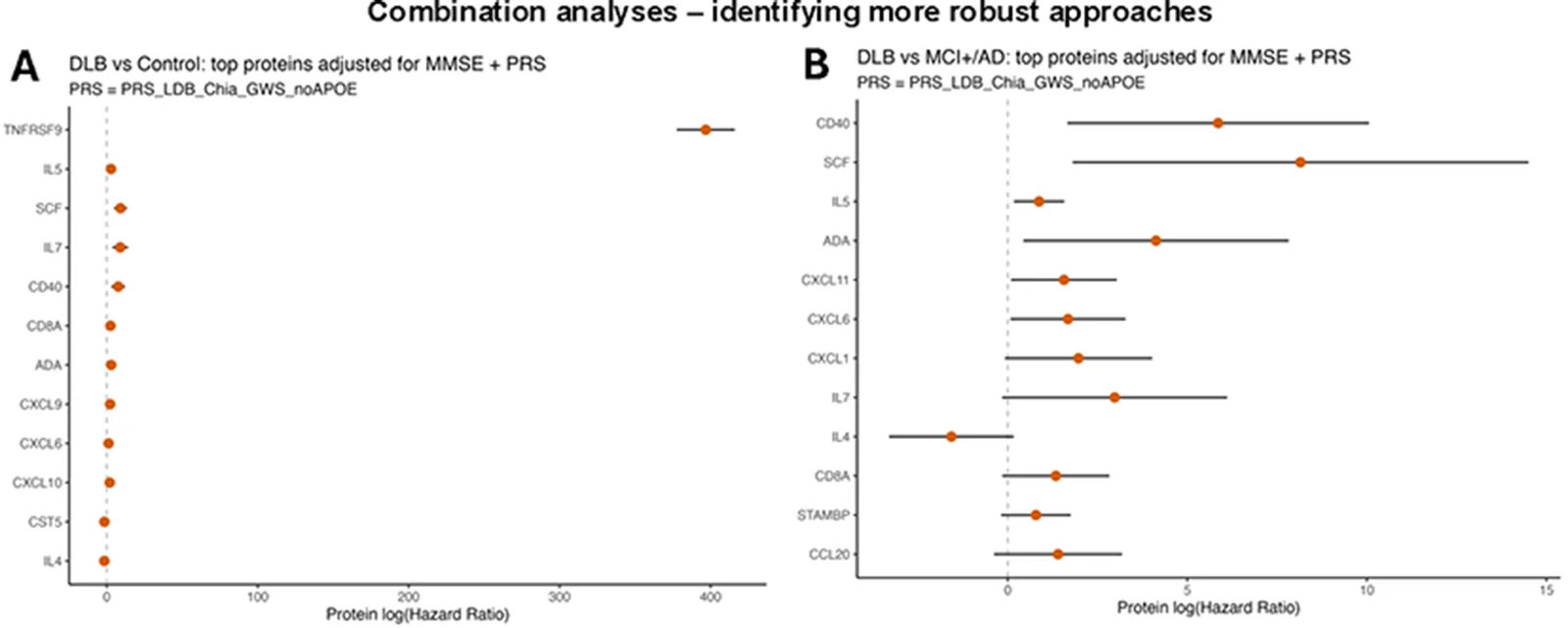
**(A)** Forest plot of selected inflammatory proteins displaying estimated effect sizes on clinical outcome, shown as log(hazard ratio). Each point represents the model-derived estimate for an individual analyte, with the dashed vertical line indicating the null effect (log(HR) = 0). Notably, TNFRSF9 exhibits a markedly elevated effect size relative to other proteins. **(B)** Expanded forest plot of top-ranked proteins with corresponding confidence intervals, highlighting both directionality and precision of estimated effects. Points denote log(hazard ratios), with horizontal lines representing 95% confidence intervals. Several proteins, including CD40, SCF, and ADA, demonstrate positive associations with outcome measures, whereas IL-4 trends toward a negative association. Chemokines (e.g., CXCL11, CXCL6, CXCL1) and interleukins (e.g., IL-5, IL-7) show moderate positive effects.

**Table 1:** Participant demographics for *Olink* Inflammatory Panel.

Variable	Control (n = 15)	MCI+/AD (n = 15)	DLB (n = 20)	p-value
Age	68.8 ± 5.5	72.5 ± 9.2	72.8 ± 6.0	0.20423887
Sex	M7/F8	M11/F4	M17/F3	0.089576085
MMSE	28.9 ± 1.1	25.7 ± 2.4	22.4 ± 4.5	2.8803E-06
ACE-R	92.1 ± 5.6	79.8 ± 8.1	67.2 ± 13.4	3.6652E-08
Survival in years (mean)	-	6.42 (n = 15, 5 deaths)	3.89 (n = 20, 17 deaths)	< 0.0001

## Data Availability

The scripts utilized for analysis and presented in this study are available on request from the corresponding author and data is available in the Supplementary File 1 provided.
